# The Treatment of Fractures from a Common-Sense Point of View

**Published:** 1907-12-07

**Authors:** 


### Points in Surgery.

### THE TREATMENT OF FRACTURES FROM A COMMON-SENSE POINT OF VIEW.

### XV.—FRACTURES OF THE TIBIA AND FIBULA.

Fracture of the shaft of the tibia is one of the commonest of major surgical injuries. In most cases the accident is caused by a blow directly applied to the shin, such as a kick, or by the passage of a vehicle over the leg. Moreover, owing to the density of the shaft of the tibia and its angular contour, the line of the fracture is rarely a simple transverse one, and it is here that one meets with the varieties to which special names have been given, namely spiral, oblique, and *en bec de flute*. When the leg is inspected little or no displacement may be seen, especially if, as sometimes happens, the line of fracture is transverse; as a general rule, the more oblique the line is, the more displacement will there be. Most often the upper end of the lower fragment is driven backwards, probably as the result of the continuation of the direction of the force which caused the accident. Far less commonly it is carried forwards, and its sharp upper extremity may perforate the skin and convert a simple into a compound fracture. It is quite the exception to find the lower fragment dragged up on the upper, so that it is rarely necessary to employ any form of extension in these cases. If extension has to be employed, it must be applied from a boot, because there is not a sufficient area of the limb below the fracture for the application of the ordinary extension apparatus. Some authorities advise one to divide the tendo Achillis if any difficulty is experienced in reducing the displacement. But the advice is not sound. The operation considerably lengthens the period of convalescence, and may leave the patient with a seriously weakened leg. The writer has no experience of a case in which complete reduction could not be effected by patient manipulation under an anæsthetic.

In a straightforward case the treatment is comparatively simple. After reducing any displacement the limb should be put on a padded back splint and two side splints. One word of advice about the back splint: these splints are commonly made in two patterns; in one the foot is at right angles to the back, but in the other it is set at a broader angle, so that the limb is put up with some degree of plantar flexion at the ankle-joint. There is no doubt that the latter is the more comfortable of the two, but it should not be used if the fracture is in the lower third of the limb, for the following reason: The anterior part of the upper articular surface of the astragalus is broader than the posterior. If the bones are allowed to unite with the foot in the position of plantar flexion, the malleoli, which are only now separated by the narrow posterior part, become permanently approximated, so that when the patient begins to get about he finds that he cannot bring his foot up to a right angle, and some degree of talipes equinus results. Massage and passive movement should be begun early, and after a varying time, which must be judged in each individual case, generally about two weeks, the limb may be put up in a Bavarian splint, which reaches to above the knee. Union should be firm enough to allow the patient to begin to get about on crutches in six weeks. There are several troublesome complications which have to be dealt with. One is the tendency of the upper end of the lower fragment to come forward while the leg is on the splint owing to the weight of the foot. This not only causes irregularity in the line of the bone, but may damage the nutrition of the skin at that point owing to pressure. The best way to counteract this is to put a stocking on the foot. The heel is elevated by pulling on the toe of the stocking, which is then fixed to the footpiece with a tin tack or some similar contrivance. Another point of no little difficulty is the question what line of treatment is to be adopted in those cases of fracture of the tibia which are associated with a tiny punctured wound, from which blood is oozing. Is one to treat them as compound fractures, open up the wound, and thoroughly irrigate the tissues with antiseptic solutions? or is one merely to make the surface as aseptic as possible, apply a gauze dressing, and treat the case as a simple fracture? Of course, by adopting the latter alternative one runs a certain amount of risk. But, on the other hand, if the wound does show signs of becoming septic one can always open it up later; whereas if it is not infected the patient's convalescence is appreciably shortened. Again, there is often a great deal of effusion in these injuries, and since the bone is so superficial the vitality of the skin over it may be impaired, and a localised gangrene may result. In other cases blisters containing blood-stained fluid make their appearance. Either condition is, of course, a contra-indication to putting the limb up in any apparatus which prevents the skin being frequently examined and dressed. Since the damaged skin is nearly always on the front of the leg, a back splint and two side splints, loosely applied, will meet the needs of the case, but should the whole circumference be involved it is better to keep the limb in a rolled junk for a day or two until the condition of the surface begins to show improvement.

Fractures of the fibula alone are of interest, because of the difficulty of diagnosing their presence without a skiagram. In some cases the patient can walk about with a fractured fibula. It is said that after fracture there is loss of the normal spring which is transmitted to the hand on compressing the leg. This is hard to estimate. Another test consists of compressing the shaft of the fibula with one hand while the index finger of the other is applied to the apex of the external malleolus. Normally this is deflected outwards. If the deflection does not take place the fibula is broken. In these cases there is little effusion, and the skin is not involved. They are best treated by putting the limb up at once in a Bavarian splint.

